# Assessing the Impact of IOS Scanning Accuracy on Additively Manufactured Occlusal Splints

**DOI:** 10.3390/dj12100298

**Published:** 2024-09-24

**Authors:** Eduardo Anitua, Asier Lazcano, Asier Eguia, Mohammad Hamdan Alkhraisat

**Affiliations:** 1 University Institute for Regenerative Medicine and Oral Implantology—UIRMI (UPV/EHU-Fundación Eduardo Anitua), 01007 Vitoria, Spain; aeguiadelvalle@gmail.com (A.E.); dr.khraisat@gmail.com (M.H.A.); 2 BTI Biotechnology Institute, 01005 Vitoria, Spain; asier.lazcano@bti-implant.es; 3 Biomedical Research, Universidad del País Vasco/Euskal Herriko Unibertsitaea (UPV/EHU), 48940 Leioa, Spain; 4 Faculty of Medicine and Nursing, Universidad del País Vasco/Euskal Herriko Unibertsitaea (UPV/EHU), 48940 Leioa, Spain; 5 Faculty of Dentistry, University of Jordan, Amman 11942, Jordan

**Keywords:** nightguard, intraoral scanner, accuracy, trueness, precision, digital dentistry

## Abstract

**Introduction:** Digital workflow and intraoral scanners (IOSs) are used to clinically obtain data for a wide range of applications in restorative dentistry. The study aimed to compare two different IOSs with inexperienced users in the digital workflow of oral split manufacturing. **Material and Methods:** Anonymous stone models of upper and lower dentate patients were used. Both models were scanned with a desktop 3D scanner 3Shape D2000 to obtain the reference models (STL_R_). Ten inexperienced operators scanned each model three times with each IOS system (3Shape TRIOS 3 and Carestream CS 3800). Finally, 20 intraoral scanners were randomly chosen from the obtained dataset (10 per IOS system) to design and manufacture 20 nightguards. All the nightguards were scanned. Trueness and precision were calculated and compared between the two IOS systems. **Results:** All the mean errors both for trueness and precision were below 40 µm, more than acceptable for the design and manufacturing of intraoral devices such as nightguards. All the mean errors (except one) for trueness between the inner part of the nightguards and the upper control model were below 100 µm, less than a printed layer height. For inexperienced operators, both IOSs are suitable for a digital workflow of manufacturing occlusal splints.

## 1. Introduction

The occlusal splint is a removable intraoral appliance that clinicians use to treat a wide variety of conditions, including bruxism, temporomandibular joint disorders, and masticatory system disorders [1,2]. The occlusal splint, due to its increased surface area, decreases the stress on teeth and restorations, thus preventing mechanical-induced damage to these structures. There are different factors that influence the clinical efficacy of these types of appliances. The primary factor that determines the occlusal splint’s function is its design. Nowadays, there are different kinds and designs that have been tailored by dictating the degree of teeth coverage (partial or complete), elasticity (hard or soft), optical properties (transparency), and stability [1,2]. The material choice would additionally affect the polishability and cleansibility of the occlusal splint. Patient comfort and adherence to treatment are factors that need to be considered to ensure an effective treatment [3].

Additive manufacturing (AM) has been suggested and implemented to produce occlusal splints. This manufacturing method is based on a three-dimensional digital design of the occlusal splint (CAD) and is then printed layer by layer (CAM) [3]. The biggest advantage of AM is the reduction of the labor load by scaling up the production capacity and reproducibility. Furthermore, it allows for the incorporation of artificial intelligence in the design process to tailor it to clinical efficacy and safety. Salmi et al. have reported reduced chair-side time due to the use of the occlusal splint that was fabricated by AM [3].

The first step in the AM of the occlusal splint is the digitalization of the teeth, gingival tissue, and maxilla–mandible relation. Nowadays, this step is widely performed by extraoral scanning of cast models or the incorporation of intraoral scanners. Intraoral scanners are classified by their accuracy and ergonomics. The more accurate an intraoral scanner is, the better results we obtained in AM solutions. Accuracy consists of two parameters: trueness and precision. Trueness is how closely the data conform to reality (or the best approximation of reality), while precision is how closely the data conform to each other, in a continuous measurement of data [4]. Therefore, for high accuracy, a scanner must have high trueness values (decreasing systematic errors) and high precision values (decreasing random errors). The combination of both leads to a high accuracy (decreasing uncertainty) [5]. Although different studies have compared different types of intraoral scanners, there is a need to assess the impact of these differences on the production of occlusal splints [6]. Patient anatomy, CAD design, manufacturing technology, and the type of material are factors that may directly impact the result of the product.

The purpose of this study has been to analyse the behaviour of 3Shape (Copenhagen, Denmark) TRIOS 3, and Carestream CS 3800 (Atlanta, GA, USA) by inexperienced operators in a full arch dentate model that was going to be used for nightguard (intraoral devices) design and manufacturing. The null hypotheses were that no significant difference would be found between 3Shape TRIOS 3 and CS Carestream CS 3800 accuracy (trueness and precision), and that no significant deviation would be added in occlusal splint manufacturing and post-processing.

## 2. Materials and Methods

### 2.1. Reference Model and Scanning Procedure

Anonymous stone models of upper and lower dentate patients were used for the study. Both maxilla and mandible did not present any dental restoration and had ideal occlusal plane. It was a Class I dentate patient, resulting in models that are easy to scan due to their low complexity. Then, reference file was generated to be used as a control for the comparison with the intraoral scanners. Two types of intraoral scanners were used 3Shape TRIOS 3 and Carestream CS 3800. Reference model: The working casts were digitized to serve as references or control models in the study by means of a Blue LED Multi-line structured light (D2000; 3Shape) and four cameras of 5MP. This device projects a series of parallel patterns onto the scan target. When light patterns are projected onto the plaster model, the patterns become distorted. The four cameras capture these visualizations from different angles and process them to find the best possible point cloud reconstruction, obtaining the digitization of the plaster models as STL files named control or reference (STL_R_). The D2000 3Shape scanner had been previously calibrated following the manufacturer’s manual, reporting an accuracy of 5 µm (ISO 12836). The calibration process is done by inserting a calibration object without touching the top surface. Scanned data were reported to be sufficiently accurate for use as a reference model.

**Figure 1:**
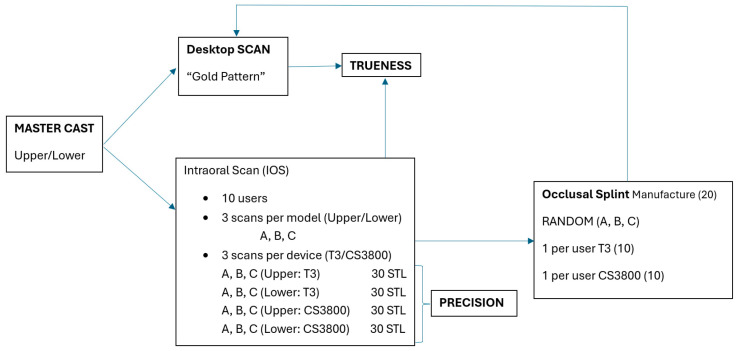
Schematic representation of the study design.

**Figure 2:**
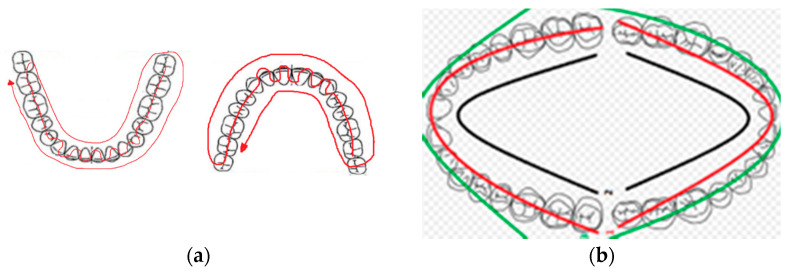
(**a**) Scanning protocol for the maxillary complete-arch digital scans performed followed 3Shape recommendation; (**b**) scanning protocol for the maxillary right quadrant digital scans performed followed Carestream recommendations.

Intraoral scan (IOS) models: Ten unexperienced odontologists performed digital scans of the cast models (maxilla, mandible), three times each for each model with each IOS. The odontologists were identified by numbers (from 122 to 131) to maintain their anonymity. Everyone had at least 3 years of experience in the industry but less than a month in the digital workflow. The number of times was named as follows: A = first-time scanning, B = second-time scanning, and C = third-time scanning (Figure 1). The IOS models were calibrated following the manufacturer’s calibration protocol by inserting the IOS inside a calibration tip. Each operator scanned following the manufacturer’s scanning protocols. Further, 3Shape recommendation is as follows: Start the scanner while it rests occlusal on the molar and wait three-to-five clicks. Move towards the centrals, capturing the occlusal surface. Continue slowly during the centrals and again continue along the occlusal surface until you reach the last molar. Turn buccal slowly by rotating the scanner 60–90 degrees at the last molar and complete the buccal swipe, taking care of areas where soft tissue may interfere with the scan. Go along the buccal side until the last molar on the opposite side is reached. Finally, roll to the palate side and complete the swipe (Figure 2a). Carestream recommendation is as follows: Start the scanner while it rests occlusal on the molar and move along the arch scanning the occlusal surface until you reach the last molar. Turn buccal slowly by rotating the scanner 60–90 degrees at the last molar and complete the buccal swipe, taking care of areas where soft tissue may interfere with the scan. Finally, turn to the palate side and complete the arch scanning (Figure 2b).

### 2.2. Data Analysis and Assessment of Trueness and Precision

**Figure 3:**
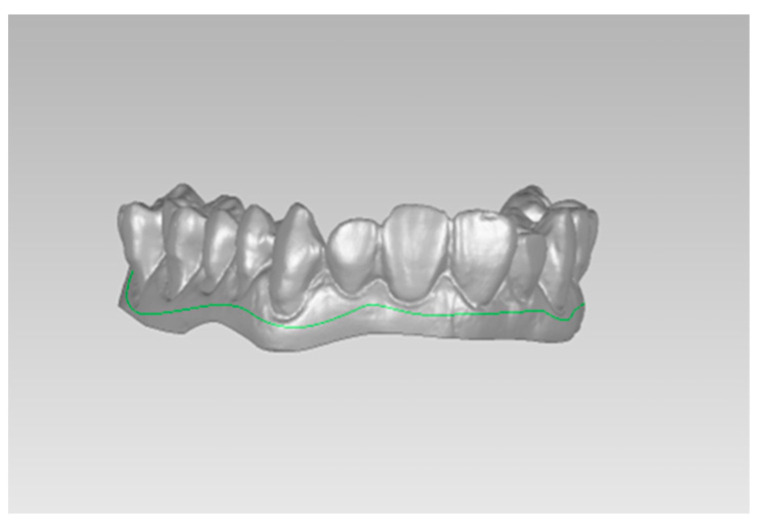
Selected ROI (in green) defined by a spline in Geomagic Studio 12.

The STL_R_ files were used as a reference digitized model to compare the deviation with the 120 files obtained by the scanning of the operators. In the study, trueness was defined as the mean distance (mm) between the reference model and each scanned model, and it was represented in a colour spectrum. Precision was defined as the mean distance deviation between scan pairs of the same operator. A 3D mesh processing software (Geomagic Studio 12) was used for 3D mesh processing. All the obtained meshes were pre-processed by inspecting the obtained tessellated geometry, cleaning and defining the region of interest (ROI) for the deviation analysis (green line):

To maintain the same ROI for all the datasets, they were first translated into the same coordinate system, and then a spline was defined between the boundary of gum and teeth (Figure 3). All the scanners were between the range of 210,000–220,000 triangles.

Regarding trueness, 60 obtained meshes from the operators were translated to same coordinate system, applying a transformation matrix 4 × 4. This matrix was obtained by means of mesh-to-mesh point-based registrations. It consists of the re-orientation of two or more objects (point or polygon) that comprise a single object so that identical regions of different objects are made to overlap. N-point registration mode type has been used, where objects were aligned on the selection of three-to-nine points on the overlapping section of the objects.

Once the objects were overlapped and located at the same coordinate system, a fine registration was applied to reduce the deviation between the two meshes. This fine registration was made by applying an algorithm known as iterative closest-point (ICP) algorithm [7]. This algorithm minimizes the difference between two sets of points. Once both meshes have been overlapped at their maximum, the difference between two surfaces (control mesh and new measured mesh) was calculated by taking the greatest of all the distances from a point in one set to the closest point in the other set, known as Hausdorff distance. The Hausdorff distance is the maximum distance from any point on either surface to the nearest point on the other. Hausdorff distance, named after the German mathematician Felix Hausdorff, measures the dissimilarity between two sets. Specifically, it measures how far two subsets of a metric space are from each other. This concept was applied in 3D meshes, which are collections of vertices and their connections (usually edges and faces) in three-dimensional space. The concept remains the same as in the general definition but is adapted to work with these mesh structures. With two 3D meshes *M*_1_ and *M*_2_, the Hausdorff distance [8,9] between them can be defined as follows:

(1) $$Hausdorff\left(M_{1},M_{2}\right)=max(\underset{v_{1}\in \;M_{1}}{\max}\underset{v_{2}\;\in \;M_{2}}{\min}\left\|v_{1}-v_{2}\right\|,\;\;\underset{v_{2}\in \;M_{2}}{max}\;\underset{v_{1}\in \;M_{1}}{min}\left\|v_{2}-v_{1}\right\|\;)$$

For each vertex *v*_1_ in *M*_1_, the minimum distance to any vertex *v*_2_ in *M*_2_ was found, and the maximum of all these minimum distances was taken, where || *v*_1_ − *v*_2_ || denotes the Euclidean distance between vertices *v*_1_ and *v*_2_. Similarly, for each vertex $v_{2}\;$in $M_{2}$, the minimum distance to any vertex *v*_1_ in $M_{1}$ was found, and the maximum of all these minimum distances was taken, where || *v*_2_ − *v*_1_ || denotes the Euclidean distance between vertices *v*_2_ and *v*_1_. The overall Hausdorff distance between the two 3D meshes is then the maximum of the two computed values.

**Figure 4:**
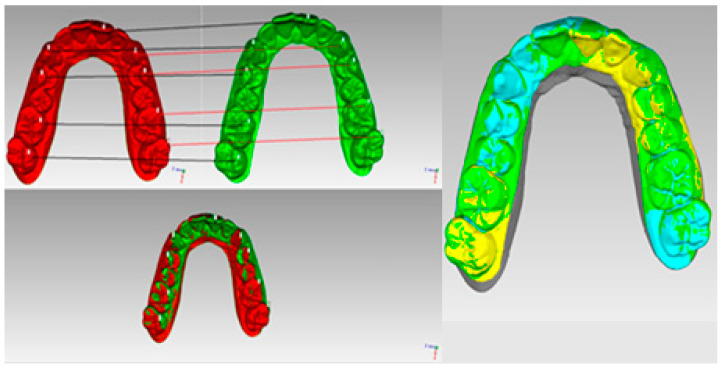
Mesh-to-mesh point-based registration, ICP algorithm, and colour-coded surface mapping.

Once the deviation values were obtained, they were plotted as a colour-coded surface mapping of the dental scan, indicating the positive and negative dimensional differences. A positive value in dimensional difference was denoted by a red–yellow spectrum on the color-coded surface map. If there was no significant dimensional difference, it was defined by green, and a negative value in dimensional difference was indicated by a blue spectrum on the colour-coded surface map. For this representation, the vertices of the mesh were labelled with the corresponding RGB colours [10,11,12,13,14]. A histogram was plotted per each analysis to see the distribution of all the cloud points (Figure 4).

Regarding precision, each 3-scan data obtained from each operator were compared in pairs, obtaining 3 sets of analysis results. The 3D deviation analysis was performed using the Hausdorff distance, which was calculated between two surfaces: AB, AC, and BC. The same process as trueness was applied, but instead of comparing the obtained STL to the control mesh, the obtained meshes were compared between them. The normality of the data distribution for the quantitative variables was tested by Shapiro–Wilk test. The standard error and the 95% confidence interval were calculated. The comparison between the study groups was performed with two-way ANOVA test. The statistical analysis was performed in IBM SPSS Statistics (Armonk, NY, USA). The threshold of statistical significance was at *p*-value < 0.05.

### 2.3. Manufacturing and Post-Processing of Nightguards for Trueness Evaluation

Two hundred forty analyses were made in total with the 120 obtained digital models: 120 for trueness and 120 for precision. From the 120 models, 20 random meshes were chosen for design and manufacturing of their corresponding nightguards. The randomicity consisted of choosing one of the three scanners made from each operator and each system, naming them with the operator number and a letter T for TRIOS 3 and C for CS 3800. These nightguards were designed by the software BTI 3D INTRAORAL DEVICES version 2.4.10, developed internally for design and manufacturing of nightguards and intraoral device APNia.

All the nightguards were manufactured by DLS technology (with a 75 µm pixel size) and biocompatible class IIa resin material (printed at 100 µm layer height), and they were labelled by their mesh *stl* name. The manufactured parts were post-processed under inner conditions (N_2_) following the material’s manufacturer protocol to avoid the creation of an oxygen inhibition layer. The inner part of the 20 splints was scanned using the reference scanner 3Shape D2000. Moreover, the obtained digitized splint models were compared to the control model STL_R_ to see the trueness between the inner part of the nightguard and the control model. To make this possible, the 20 scanned parts were translated to the same coordinate system, and an ROI was applied to all of them (the inner part of the splint). The ROI was defined by a splint, and it was extracted from the STL to obtain a new object to work on it. The obtained 20 ROIs were translated to the 3D coordinate system of the control mesh, and then ICP algorithm was applied for a best fit between control mesh and the inner part of the splint. Finally, the Haussdorf distance was applied to calculate the deviation between objects. These results showed deviation in the manufacturing process, and they were supposed to have higher deviation than the scanned models due to the deviations that may occur in the manufacturing and post-processing processes.

## 3. Results

Two hundred forty analyses were conducted for trueness and precision. In all groups, the standard deviation was higher than the mean errors from the control mesh. All of the mean errors, both for trueness and precision, were below 40 µm, indicating that the average distance was anywhere between 210,000–220,000 triangles of each scanner, and the control model and each scanner, as well as the following ones (A, B, and C) done by each operator, was below 40 microns, more than acceptable for the design and manufacturing of intraoral devices such as nightguards [13].

Regarding the trueness of the upper model scanned by TRIOS 3 (Table 1), mean values mostly ranged between −0.05 mm and 0.05 mm. Slight deviations were observed, but most data points were concentrated around 0 mm. Standard deviations were relatively consistent across times A, B, and C, with a general range from 0 to 0.1 mm. Maximum positive deviations showed some outliers, but most values were clustered below 1.5 mm. Maximum negative deviations had a few outliers extending below −1 mm, with most values above −1.5 mm.

Regarding the trueness of the lower model scanned by TRIOS 3 (Table 2), mean values were tightly grouped around 0 mm, with occasional slight positive and negative deviations. Standard deviations ranged similarly to the upper model, generally between 0 and 0.1 mm. Maximum positive deviations showed a similar pattern with most values below 1 mm. Maximum negative deviations had more outliers, with values occasionally dropping below −1 mm.

With respect to the trueness of the upper model scanned by CS 3800 (Table 3), negative mean deviations were more common, with most values within −0.1 to 0.05 mm. Standard deviations were slightly higher, reaching up to 0.15 mm. Maximum positive deviations were generally below 1.5 mm but showed some significant outliers. Maximum negative deviations extended significantly, with some values going below −2 mm.

Concerning the trueness of the lower model scanned by CS 3800 (Table 4), mean deviations showed more variation, both positive and negative, with a similar range as the upper model. Standard deviations were higher on average, with more outliers. Maximum positive deviations reached higher values with several significant outliers. Maximum negative deviations showed a broader range with more extreme outliers.

Relating to the precision of the upper model scanned by TRIOS 3 (Table 5), mean deviations were relatively small, mostly within ±0.05 mm. Standard deviations were consistent and low, mostly under 0.05 mm. Maximum positive deviations were mostly under 0.5 mm. Maximum negative deviations were generally under −1 mm, with some outliers.

About the precision of the lower model scanned by TRIOS 3 (Table 6), mean deviations were small and centered around 0 mm. Standard deviations were consistently low. Maximum positive deviations remained low, under 0.5 mm. Maximum negative deviations showed a few outliers but were generally under −1 mm.

With respect to the precision of the upper model scanned by CS 3800 (Table 7), mean deviations were slightly larger, with more variation. Standard deviations were higher and showed more outliers. Maximum positive deviations had higher values, with significant outliers. Maximum negative deviations showed more variation, with values extending further.

Regarding the precision of the lower model scanned by CS 3800 (Table 8), mean deviations were small and around 0 mm but showed more negative values. Standard deviations were low but had more variation. Maximum positive deviations showed higher values and more outliers. Maximum negative deviations had significant outliers and broader ranges.

All results showed that the operators’ experience had little impact on the trueness and precision of the study. In the trueness of TRIOS 3 (Table 9), mean deviations across operators were relatively small and centered around 0 mm, indicating that most operators were able to achieve high trueness. Standard deviations were consistently low across operators, suggesting good repeatability and precision in measurements. Max deviations showed some outliers, but these were not significantly different across operators, indicating that extreme values were likely due to measurement challenges rather than operator inconsistency. In the trueness of Carestream CS 3800 (Table 10), mean deviations varied more among operators, indicating that some operators might have had difficulties achieving high trueness consistently. Standard deviations were higher on average and showed more variability among operators, suggesting that some operators were less consistent in their measurements. Max deviations had more significant outliers, which could be linked to specific operators struggling with certain measurements.

Regarding the precision of TRIOS 3 (Table 11), mean deviations were small and consistent across operators, indicating high trueness between them. Standard deviations were low and consistent, suggesting high precision across operators. Max deviations showed fewer outliers, indicating fewer instances of significant measurement errors among operators. With respect to the precision of CS3800 (Table 12), mean deviations varied more significantly among operators, suggesting differences in measurement techniques or challenges with the equipment. Standard deviations were higher and more variable among operators, indicating inconsistencies in measurements. Max deviations had more significant outliers, pointing to occasional large errors that could be operator-dependent.

Regarding the precision of the IOS, in the mean deviation of lower arch models, there were statistically significant differences, as the *p*-value was 0.032. However, in the mean deviation of upper arch models, there were no statistically significant differences, as the *p*-value was 0.921. Regarding trueness, in the mean deviation of lower arch models, there were statistically significant differences as the *p*-value was 0.000 and in the mean of upper models.

The manufacturing and post-processing processes of polymers also had a great impact on the deviation of the final product in the digital workflow. Standard deviations for trueness between the inner part of the nightguards and the upper control model were higher than the mean errors from the control mesh. All the mean errors for trueness were below 100 microns, except one that was 244 microns. This last one could be affected by the holder of the scanning parts. All the nightguards were placed in a holder inside the scanner, and due to the low Young modulus of the polymer (1063 MPa at environmental temperature), it could suffer some deformation when scanning. However, due to the low stiffness of the material, the deviation suffered in the manufacturing and post-processing processes could be corrected when placing the nightguard in a patient’s mouth. This was a good point because it was possible to quantify all of the possible errors in the digital workflow of the design and manufacturing of nightguards with DLS printing technology and the biocompatible Class ii material that was used in the study (Table 13).

**Figure A1:**
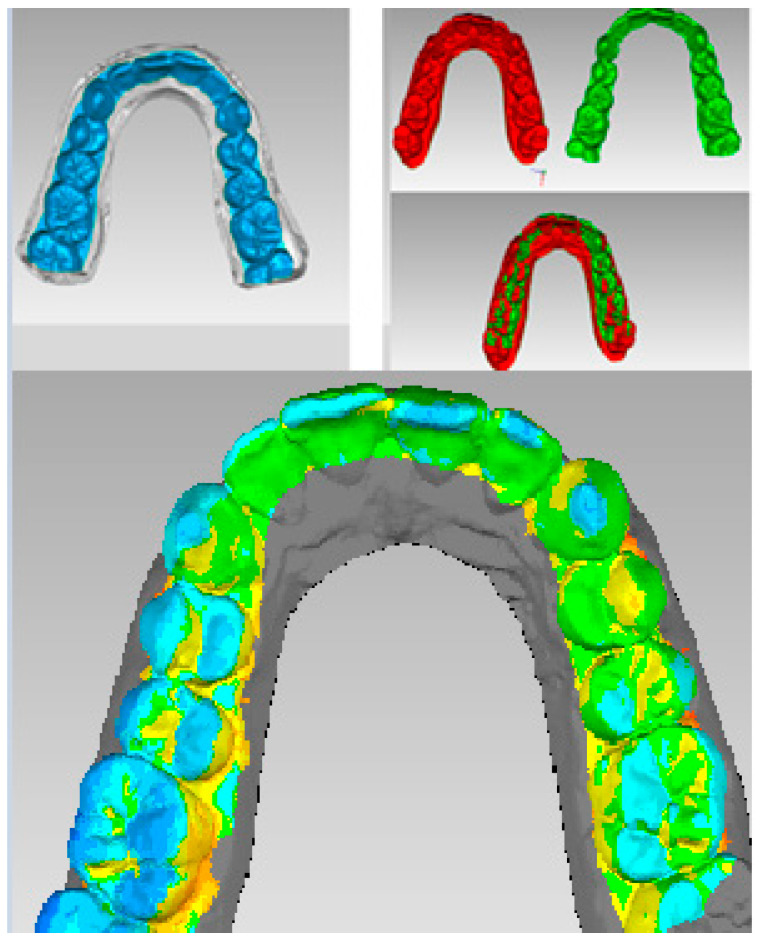
Mesh-to-mesh point-based registration, ICP algorithm, and colour-coded surface mapping of the manufactured splints and the STL_R_ model.

The mean deviations for different splints were relatively small, with values centered around 0 mm. This indicated good trueness overall, as the deviations from the true value are minimal. Also, 122C and 123T splints showed slightly larger mean deviations compared to others, suggesting some variability in the measurements. The other splints generally showed minimal mean deviations, indicating high trueness. The standard deviations were low across all splints, indicating consistent measurements with minimal spread around the mean. Further, 122C and 123T showed slightly higher standard deviations, indicating more variability in the measurements for these splints. The other splints consistently obtained low standard deviations, indicating precise and repeatable measurements. Positive maximum deviations were generally below 2 mm, with a few outliers extending beyond this range. Also, 122C showed the highest positive maximum deviation, indicating occasional significant positive errors. The most positive deviations of the other splints were clustered below 1.5 mm, indicating fewer significant positive errors. Negative maximum deviations showed more variation, with some values extending below −2 mm. Also, 122C again showed the highest negative maximum deviation, indicating occasional significant negative errors. The other negative deviations of splints were generally less extreme, clustered above −1.5 mm (Figure A1).

## 4. Discussion

### 4.1. Impact of Digital Workflow and Scanner Accuracy in Clinical Dentistry

Digital workflow is growing exponentially in the dental sector. Although it is supposed to be a more precise technology than conventional workflow, we must consider the deviations that may occur along the workflow. In clinical dentistry, intraoral scanners (IOSs) play a vital role in obtaining digital data of the patient’s mouth for a wide range of applications such as diagnosis or restorative dentistry. Ensuring the accuracy of intraoral scanning is crucial and is determined by two key parameters: precision and trueness. Precision shows how similar repeated measurements are, in other words, the reproducibility of the scanning results of the same anatomical structure, while trueness shows how it is a similar measurement to the value of the measured anatomy. Both 3Shape TRIOS 3 and Carestream CS 3800 are suitable for intraoral devices such as nightguards. The manufacturing and post-processing methods add deviation in the digital workflow. However, due to the low stiffness of the polymer, these small deviations are not critical because it adapts perfectly when the application is placed in the mouth. The flexibility that the polymer offers can lead to the correction of these small deviations.

Other previous studies evaluated the influence of the ambient light scanning conditions on the accuracy of different IOS that have not been considered in this study [15]. The results of this study show the accuracy of just one patient model that did not present any dental restorations, so all the teeth were perfectly aligned among the arch. The number of analyses demonstrates the effectiveness of this study because 240 deviation analyses were performed, more than what had been analysed in other previous studies.

### 4.2. Comparison of Operating Technologies: Precision, Trueness, and Software Performance

In reference to the operating technologies across all models and metrics, there was a consistent trend of small mean deviations centered around 0 mm, with occasional outliers. The standard deviations were relatively low, indicating precise measurements, though a few models showed higher variation. Both positive and negative maximum deviations showed more significant outliers, indicating occasional larger discrepancies. In general, the comparative performance of the operating technologies was as follows: TRIOS 3 models generally had lower deviations and fewer outliers compared to the CS 3800 models, indicating higher precision and trueness. The results of the mean deviation of models were statistically significantly different in general, as the obtained *p*-values were as follows: 0.032/0.921 for precision and 0.000/0.000 for trueness. So, the differences were considered significant. Consequently, the first null hypothesis was rejected. In general, 3Shape TRIOS 3 results were slightly better than CS Carestream 3800, as the precision obtained between the different scanning times is very high. Regarding the tessellation of the obtained meshes, the ones obtained by TRIOS 3 were more uniform and equitable compared to the ones obtained by CS 3800. Also, small artifacts were only found in the meshes obtained by CS 3800. This could indicate that 3Shape has a greater capacity to process the data obtained from the point cloud, suggesting that they are superior in software development.

### 4.3. Operator Consistency

Regarding operator consistency in the study, operators who consistently achieve low mean deviations and low standard deviations contribute positively to the trueness of the measurements. Variability among operators suggests that training, technique, or experience may play a role. The equipment sensitivity also plays a role. Carestream showed more variability among operators, suggesting that the equipment might be more sensitive to operator technique or that certain operators find it more challenging to use consistently. Moreover, improving trueness may involve standardizing measurement techniques and providing additional training for operators who show higher variability in their measurements. The importance of following the manufacturer’s scanning protocol is huge, as a variety of scanning methods may vary the accuracy of results. By focusing on these aspects, it is possible to improve the overall trueness of the measurements by addressing operator-related variability. However, an operator can feel confident even without experience, as their data acquisition is valid for this type of application, as has been demonstrated.

### 4.4. Impact of Manufacturing and Post-Processing

The manufacturing and post-processing also affect the deviation of the final product. The results of this study demonstrated that the mean values obtained with IOS are lower than the ones that are produced during the manufacturing process. The accuracy values reported are questionable if the manufacturing technology [15], post-processing method, or the material is changed [16,17,18,19]. However, all the mean errors for trueness were below 100 microns (less than the manufactured layer height). So, the second null hypothesis was retained due to the insignificant deviation in the manufacturing and post-processing steps [20].

### 4.5. Study Limitations and Future Research

The results may vary depending on the tessellation geometry of the reference and test object. If decimation or refining algorithms are applied to the analysed meshes, the Haussdorf distances are modified so they can be manipulated to obtain better or worse results. In this study, the tessellation was not modified. It was used in the “raw” mesh exported from the IOS directly to avoid these possible modifications.

The limitations of the study are as follows: It was an in vitro study conducted with only one patient without prosthetic restorations, with an ideal arch and occlusal planes. The version of the intraoral scanners is not the latest in the market.

It would be interesting to analyse environmental variables such as ambient light and other models with prosthetic restorations or deteriorated teeth. Also, the study evaluates two different intraoral scanners that have different hardware components. However, it would be interesting to analyse the different optical behaviours [21].

## 5. Conclusions

- Both 3Shape TRIOS 3 and Carestream CS 3800 are suitable for creating intraoral devices like nightguards.
- Operators do not need prior experience with any IOS system to achieve successful results.
- Manufacturing and post-processing methods introduce deviations in the digital workflow.
- These small deviations are not critical due to the low stiffness of the polymer used.
- The application can be deformed in the mouth to meet the patient’s anatomical requirements.

## Figures and Tables

**Table 1:** Statistical aggregates for trueness of the upper maxilla scanned by 3Shape TRIOS 3. All values are provided in millimeters (mm).

Splint	Time	Mean	SD	Max +	Min −
122	A	0.003	0.043	0.565	−0.976
122	B	0.018	0.059	1.101	−0.926
122	C	0.007	0.041	0.298	−1.106
123	A	0.011	0.059	0.473	−1.442
123	B	0.017	0.074	0.389	−1.417
123	C	0.006	0.057	0.4	−1.488
124	A	0.008	0.047	0.314	−1.333
124	B	0.009	0.044	0.352	−1
124	C	0.01	0.042	0.312	−1.056
125	A	0.007	0.046	0.393	−1.116
125	B	0.011	0.065	0.451	−1.525
125	C	0.014	0.066	0.362	−1.395
126	A	0.006	0.039	0.45	−0.901
126	B	0.006	0.047	0.314	−1
126	C	0.004	0.044	0.288	−1.094
127	A	0.011	0.039	0.595	−0.728
127	B	−0.003	0.043	0.372	−1.004
127	C	0.001	0.042	0.381	−1.077
128	A	0.008	0.057	0.324	−1.412
128	B	0.009	0.048	0.314	−1.131
128	C	0.009	0.054	0.298	−1.367
129	A	0.008	0.041	0.308	−1.068
129	B	0.002	0.053	0.318	−1.285
129	C	0.012	0.044	0.326	−0.885
130	A	0.002	0.044	0.319	−1.343
130	B	0.005	0.037	0.291	−0.738
130	C	0.004	0.037	0.29	−0.739
131	A	0.012	0.049	0.357	−1.041
131	B	0.007	0.048	0.33	−1.248
131	C	0	0.054	0.335	−0.98

**Table 2:** Statistical aggregates for trueness of the lower mandible scanned by 3Shape TRIOS 3. All values are provided in millimeters (mm).

Splint	Time	Mean	SD	Max +	Min −
122	A	0.012	0.043	0.46	−0.633
122	B	0.02	0.06	0.724	−0.621
122	C	0.014	0.047	1.268	−0.559
123	A	0.01	0.055	0.872	−1.207
123	B	0.01	0.057	0.745	−1.247
123	C	0.01	0.057	0.8	−1.376
124	A	0.01	0.053	0.651	−1.089
124	B	0.014	0.059	0.499	−0.934
124	C	0.013	0.052	0.771	−0.947
125	A	0.009	0.061	0.675	−1.036
125	B	0.012	0.054	1.101	−1.031
125	C	0.015	0.057	1.056	−1.197
126	A	0.015	0.06	1.059	−1.432
126	B	0.013	0.069	0.542	−0.705
126	C	0.01	0.052	0.473	−0.763
127	A	0.011	0.049	0.702	−1.134
127	B	0.009	0.045	0.572	−0.664
127	C	0.007	0.045	0.446	−0.804
128	A	0.009	0.057	0.494	−0.967
128	B	0.01	0.055	1.093	−1.136
128	C	0.014	0.056	1.047	−0.947
129	A	0.015	0.057	0.724	−1.072
129	B	0.014	0.052	1.098	−1.223
129	C	0.019	0.056	0.624	−0.918
130	A	0.01	0.047	0.688	−0.69
130	B	0.009	0.049	0.608	−1.12
130	C	0.014	0.05	1.154	−0.912
131	A	0.012	0.052	0.735	−1.778
131	B	0.02	0.053	1.069	−1.109
131	C	0.016	0.056	1.025	−1.12

**Table 3:** Statistical aggregates for trueness of the upper maxilla scanned by Carestream CS 3800. All values are provided in millimeters (mm).

Splint	Time	Mean	SD	Max +	Min −
122	A	−0.032	0.075	0.412	−0.857
122	B	0.007	0.056	0.419	−1.069
122	C	−0.016	0.065	0.572	−1.204
123	A	−0.017	0.068	1.12	−0.912
123	B	−0.008	0.06	0.41	−0.824
123	C	−0.019	0.096	0.449	−0.897
124	A	−0.01	0.07	0.486	−1.154
124	B	−0.014	0.067	0.681	−0.909
124	C	−0.032	0.099	0.475	−0.954
125	A	−0.006	0.053	0.468	−0.978
125	B	0.008	0.049	0.363	−0.972
125	C	0.006	0.05	0.391	−0.938
126	A	−0.002	0.057	0.408	−0.924
126	B	−0.001	0.063	0.401	−0.944
126	C	−0.008	0.063	0.559	−1.016
127	A	−0.02	0.059	0.413	−0.896
127	B	−0.02	0.059	0.407	−1.29
127	C	−0.021	0.066	0.48	−1.035
128	A	−0.002	0.059	0.455	−0.859
128	B	0.004	0.062	0.441	−1.026
128	C	0.008	0.066	0.439	−0.949
129	A	0	0.051	0.382	−1.051
129	B	0.005	0.057	0.483	−1.062
129	C	0.007	0.085	0.431	−1.232
130	A	0.008	0.062	0.783	−0.962
130	B	−0.004	0.057	0.494	−1.206
130	C	−0.005	0.055	0.475	−0.902
131	A	0.009	0.053	0.328	−0.784
131	B	0.002	0.056	0.479	−0.939
131	C	0.002	0.053	0.427	−1.097

**Table 4:** Statistical aggregates for trueness of the lower mandible scanned by Carestream CS 3800. All values are provided in millimeters (mm).

Splint	Time	Mean	SD	Max +	Min −
122	A	−0.008	0.086	1.039	−3.053
122	B	−0.012	0.083	0.913	−1.027
122	C	−0.013	0.099	0.859	−1.267
123	A	0.011	0.09	0.755	−0.97
123	B	−0.02	0.089	1.2	−0.907
123	C	0.009	0.088	0.688	−0.757
124	A	−0.013	0.072	0.804	−1.105
124	B	−0.003	0.061	1.168	−1.219
124	C	−0.005	0.075	0.772	−0.904
125	A	−0.003	0.068	1.036	−1.097
125	B	−0.002	0.057	0.553	−0.926
125	C	−0.003	0.075	0.851	−0.995
126	A	0.001	0.065	0.847	−0.756
126	B	0	0.067	0.608	−0.783
126	C	−0.003	0.071	0.404	−1.277
127	A	−0.008	0.066	0.477	−0.849
127	B	−0.001	0.072	0.899	−1.159
127	C	−0.018	0.068	0.456	−0.935
128	A	0.01	0.057	0.49	−0.965
128	B	0.01	0.055	1.094	−1.137
128	C	0.013	0.056	1.044	−0.948
129	A	0.015	0.057	0.724	−1.072
129	B	0	0.08	1.124	−0.878
129	C	−0.016	0.075	1.122	−1.179
130	A	−0.002	0.071	0.947	−1.03
130	B	−0.007	0.074	0.895	−0.992
130	C	−0.001	0.065	1.084	−0.84
131	A	0.008	0.072	0.88	−1.138
131	B	0	0.07	1.091	−1.088
131	C	0.005	0.065	1.036	−1.04

**Table 5:** Statistical aggregates for precision of the upper maxilla scanned by 3Shape TRIOS 3. All values are provided in millimeters (mm).

Splint	Time	Mean	SD	Max +	Min −
122	AB	0.014	0.055	1.261	−1.002
122	AC	0.004	0.029	0.177	−0.564
122	BC	−0.012	0.064	0.348	−1.522
123	AB	0.004	0.033	0.119	−0.199
123	AC	−0.005	0.014	0.081	−0.203
123	BC	−0.01	0.038	0.29	−0.178
124	AB	0.001	0.015	0.244	−0.127
124	AC	0.002	0.017	0.484	−0.217
124	BC	0.001	0.016	0.357	−0.252
125	AB	0.004	0.036	0.129	−1.327
125	AC	0.005	0.042	0.309	−1.207
125	BC	0.001	0.016	0.349	−0.434
126	AB	0	0.032	0.463	−1.06
126	AC	−0.002	0.028	0.134	−1.097
126	BC	−0.002	0.022	0.12	−0.524
127	AB	−0.014	0.052	0.434	−1.314
127	AC	−0.009	0.047	0.376	−1.41
127	BC	0.004	0.029	0.155	−0.536
128	AB	0	0.022	0.506	−0.272
128	AC	0.001	0.019	0.282	−0.217
128	BC	0	0.019	0.181	−0.486
129	AB	−0.005	0.036	0.147	−1.403
129	AC	0.003	0.024	0.901	−0.737
129	BC	0.007	0.026	1.049	−0.189
130	AB	0.002	0.016	0.661	−0.418
130	AC	0.002	0.021	0.613	−0.415
130	BC	−0.001	0.023	0.62	−0.348
131	AB	−0.005	0.031	0.353	−0.238
131	AC	−0.011	0.041	0.368	−0.236
131	BC	−0.006	0.026	0.431	−0.4

**Table 6:** Statistical aggregates for precision of the lower mandible scanned by 3Shape TRIOS 3. All values are provided in millimeters (mm).

Splint	Time	Mean	SD	Max +	Min −
122	AB	0.006	0.04	0.703	−0.448
122	AC	0.001	0.025	0.827	−0.445
122	BC	−0.007	0.044	0.738	−1.215
123	AB	0.001	0.015	0.136	−0.319
123	AC	0.001	0.014	0.185	−0.816
123	BC	0.001	0.009	0.477	−0.572
124	AB	0.005	0.048	0.189	−0.281
124	AC	0.004	0.031	0.165	−0.409
124	BC	−0.001	0.023	0.323	−0.338
125	AB	0.005	0.025	0.229	−0.642
125	AC	0.006	0.05	0.234	−0.569
125	BC	0.001	0.029	0.192	−0.401
126	AB	−0.003	0.025	0.534	−0.384
126	AC	−0.004	0.058	0.539	−0.201
126	BC	0	0.076	0.459	−0.344
127	AB	−0.002	0.024	0.691	−0.911
127	AC	−0.004	0.024	0.415	−0.774
127	BC	−0.003	0.033	0.599	−0.555
128	AB	0	0.015	0.223	−0.255
128	AC	0.004	0.046	0.227	−0.251
128	BC	0.003	0.038	0.227	−0.427
129	AB	0	0.019	0.425	−0.212
129	AC	0.004	0.031	0.353	−0.163
129	BC	0.004	0.029	0.361	−0.302
130	AB	−0.001	0.015	0.331	−0.446
130	AC	0.004	0.018	0.223	−0.436
130	BC	0.004	0.023	0.302	−0.342
131	AB	0.007	0.014	0.232	−0.185
131	AC	0.003	0.023	0.149	−0.208
131	BC	−0.004	0.02	0.119	−0.212

**Table 7:** Statistical aggregates for precision of the upper maxilla scanned by Carestream CS 3800. All values are provided in millimeters (mm).

Splint	Time	Mean	SD	Max +	Min −
122	AB	0.035	0.078	0.452	−0.643
122	AC	0.012	0.064	0.76	−0.465
122	BC	−0.022	0.046	0.615	−0.969
123	AB	0.01	0.032	0.214	−0.418
123	AC	−0.001	0.1	0.323	−0.337
123	BC	−0.013	0.101	0.922	−1.49
124	AB	−0.006	0.035	0.315	−0.493
124	AC	−0.022	0.1	0.462	−0.304
124	BC	−0.018	0.086	0.459	−0.306
125	AB	0.012	0.021	0.418	−0.19
125	AC	0.009	0.046	0.646	−1.063
125	BC	−0.01	0.103	1.287	−1.412
126	AB	0	0.028	0.749	−0.536
126	AC	−0.012	0.124	1.985	−2.794
126	BC	−0.005	0.047	0.72	−0.811
127	AB	0.001	0.016	0.45	−0.643
127	AC	0	0.016	0.385	−0.385
127	BC	−0.001	0.021	1.366	−0.808
128	AB	0.007	0.027	0.259	−0.387
128	AC	0.01	0.034	0.274	−0.359
128	BC	0.002	0.021	0.181	−0.226
129	AB	0.007	0.034	0.354	−1.05
129	AC	0.009	0.076	0.276	−0.352
129	BC	0.001	0.069	0.47	−0.24
130	AB	−0.012	0.026	0.185	−0.478
130	AC	−0.015	0.04	0.468	−0.442
130	BC	−0.002	0.094	1.31	−1.599
131	AB	−0.002	0.024	0.447	−0.423
131	AC	−0.007	0.036	0.163	−0.553
131	BC	−0.001	0.018	0.358	−0.319

**Table 8:** Statistical aggregates for precision of the lower mandible scanned by CS Carestream 3800. All values are provided in millimeters (mm).

Splint	Time	Mean	SD	Max +	Min −
122	AB	−0.005	0.034	0.517	−0.84
122	AC	−0.004	0.037	0.511	−0.415
122	BC	0.001	0.032	0.743	−0.358
123	AB	−0.031	0.072	0.332	−0.463
123	AC	−0.004	0.041	0.593	−0.317
123	BC	0.027	0.063	0.597	−0.319
124	AB	0.009	0.038	0.409	−0.357
124	AC	0.007	0.04	0.518	−0.244
124	BC	−0.002	0.056	0.454	−0.566
125	AB	0.001	0.033	0.192	−0.312
125	AC	0	0.071	0.231	−1.276
125	BC	−0.001	0.053	0.383	−0.868
126	AB	−0.003	0.023	0.295	−0.63
126	AC	−0.004	0.035	0.571	−0.641
126	BC	−0.002	0.031	0.322	−0.714
127	AB	0.006	0.027	0.341	−0.475
127	AC	−0.012	0.029	0.266	−0.449
127	BC	−0.018	0.033	0.186	−0.475
128	AB	−0.013	0.038	0.373	−0.264
128	AC	−0.014	0.048	0.558	−0.707
128	BC	−0.003	0.033	0.266	−0.366
129	AB	−0.003	0.074	1.114	−0.74
129	AC	−0.017	0.047	0.391	−0.264
129	BC	−0.014	0.08	0.398	−0.41
130	AB	−0.004	0.017	0.291	−0.218
130	AC	0.002	0.025	0.323	−0.235
130	BC	0.005	0.027	0.415	−0.347
131	AB	−0.008	0.041	0.664	−0.811
131	AC	−0.003	0.036	0.221	−0.259
131	BC	0.003	0.033	0.205	−0.225

**Table 9:** Trueness 3Shape TRIOS 3.

Arch	Mean (mm)	Mean SD (mm)	Mean Max +	Mean Min −
Mandible	0.007	0.048	0.387	−1.127
Maxilla	0.012	0.053	0.792	−1.012

**Table 10:** Trueness Carestream CS 3800.

Arch	Mean (mm)	Mean SD (mm)	Mean Max +	Mean Min −
Mandible	−0.005	0.063	0.484	−0.994
Maxilla	−0.001	0.071	0.862	−1.076

**Table 11:** Precision 3Shape TRIOS 3.

Arch	Mean (mm)	Mean SD (mm)	Mean Max +	Mean Min −
Mandible	0	0.029	0.398	−0.617
Maxilla	0.001	0.029	0.36	−0.435

**Table 12:** Precision CS Carestream 3800.

Arch	Mean (mm)	Mean SD (mm)	Mean Max +	Mean Min −
Mandible	−0.001	0.052	0.575	−0.683
Maxilla	−0.003	0.041	0.422	−0.485

**Table 13:** Statistical aggregates for trueness of the nightguard. All values are provided in millimeters (mm).

Splint	Mean (mm)	SD (mm)	Max +	Min −
122C	−0.046	0.716	2.444	−2.939
122T	0	0.432	1.613	−1.72
123C	−0.013	0.297	1.318	−1.176
123T	0.005	0.474	1.534	−1.613
124C	−0.045	0.289	1.004	−1.199
124T	−0.074	0.596	2.029	−2.266
125C	−0.09	0.333	1.274	−1.601
125T	−0.012	0.482	1.822	−1.897
126C	0	0.232	1.095	−1.221
126T	−0.059	0.574	1.986	−2.248
127C	−0.091	0.856	2.933	−2.94
127T	−0.061	0.436	1.345	−1.754
128C	0	0.29	1.3	−0.951
128T	−0.244	0.706	2.936	−2.94
129C	−0.087	0.328	1.116	−1.681
129T	−0.036	0.486	1.526	−1.902
130C	0	0.17	0.672	−0.501
130T	−0.085	0.375	1.331	−1.647
131C	−0.01	0.232	0.623	−0.729
131T	0	0.134	0.736	−0.607

## Data Availability

The datasets used and analyzed during the current study are available from the corresponding author upon reasonable request.
